# Supplementary material for: Protective human IgE responses are promoted by comparable life-cycle dependent Tegument Allergen-Like expression in Schistosoma haematobium and Schistosoma mansoni infection
Source: PLoS Pathog. 2023 May 25;19(5):e1011037. doi: 10.1371/journal.ppat.1011037 (PMC10212073; doi:10.1371/journal.ppat.1011037)
Supplement: S1 Text — Fig A. Lifecycle transcription profiles of S. haematobium TAL proteins. Fig B. Lifecycle transcription profiles of S. mansoni TAL proteins. Fig C. Gel electrophoresis of purified SmTAL and ShTAL proteins. Fig D. Pre- and post-treatment (Rx) seroprevalence of IgE responses to S. haematobium TAL proteins by age and village, as a proxy for force of transmission. Table A. Percent identity matrix between ShTAL1, 3, 5 and 11 and SmTAL1, 3, 5 and 11. Table B. Recombinant antigen coating concentration. Table C. Association between pre-treatment ShTAL-specific IgE responses (seropositivity) and baseline CAA, as a measure of worm burden. Table D. Association between pre-treatment ShTAL-specific IgE responses (seropositivity) and baseline CAA, as a measure of worm burden. (DOCX) [file ppat.1011037.s001.docx]

**S1 Text**

**Liquid Chromatography Tandem Mass Spectrometry (LC-MS/MS) methodology**

**Sample preparation**

Samples were subjected to enzymatic digestion with trypsin overnight at 37°C. After digestion, the supernatant was pipetted into a sample vial and loaded onto an autosampler for automated LC-MS/MS analysis.

**LC-MS/MS**

All LC-MS/MS experiments were performed using a Dionex Ultimate 3000 RSLC nanoUPLC (Thermo Fisher Scientific Inc, Waltham, MA, USA) system and a Q Exactive Orbitrap mass spectrometer (Thermo Fisher Scientific Inc, Waltham, MA, USA). Separation of peptides was performed by reverse-phase chromatography at a flow rate of 300 nL/min and a Thermo Scientific reverse-phase nano Easy-spray column (Thermo Scientific PepMap C18, 2 μm particle size, 100A pore size, 75 μm i.d. x 50 cm length). Peptides were loaded onto a pre-column (Thermo Scientific PepMap 100 C18, 5 μm particle size, 100A pore size, 300 μm i.d. x 5mm length) from the Ultimate 3000 autosampler with 0.1% formic acid for 3 minutes at a flow rate of 15 μL/min. After this period, the column valve was switched to allow elution of peptides from the pre-column onto the analytical column. Solvent A was water + 0.1% formic acid and solvent B was 80% acetonitrile, 20% water + 0.1% formic acid. The linear gradient employed was 2-40% B in 40 minutes. Further wash and equilibration steps gave a total run time of 60 minutes.

The LC eluant was sprayed into the mass spectrometer by means of an Easy-Spray source (Thermo Fisher Scientific Inc.). All *m/z* values of eluting ions were measured in an Orbitrap mass analyzer, set at a resolution of 35000 and was scanned between *m/z* 380-1500. Data dependent scans (Top 20) were employed to automatically isolate and generate fragment ions by higher energy collisional dissociation (HCD, NCE:26%) in the HCD collision cell and measurement of the resulting fragment ions was performed in the orbitrap analyser, set at a resolution of 17500. Singly charged ions and ions with unassigned charge states were excluded from being selected for MS/MS and a dynamic exclusion window of 20 seconds was employed.

**Sequencing and multiple sequence alignment of ShTAL5 expression vector Sanger sequence results**

Multiple sequence alignment of ShTAL5 (version 1 and version 3 genome annotation) cDNA sequences with SmTAL5, SmTAL6 and results from Sanger sequencing of ShTAL5-pGEX expression vector, performed in CLUSTAL O (1.2.4). ShTAL5 V3.0 sequence truncated at 567 nucleotides.

ShTAL5_v3_MS3_00002091 ---------------------------------ATGTATCAACAAAATGAGACAATTACA 27

SmTAL5_Smp_195090 ATGGAACCATTTGTTAATATTTTTTTTGCTATAGATGAGCAACAGAATGAAACAATTACA 60

ShTAL5_v1_MS3_05957 ATGGAACCATTTGTTAATATATTTTTTGCGATAGATGAGCAACAAAATGAGACAATTACA 60

ShTAL5_expression_vector ATGGAACCATTTGTTAATATATTTTTTGCGATAGATGAGCAACAAAATGAGACAATTACA 60

.: * *****.*****.*********

ShTAL5_v3_MS3_00002091 AGAGATGAATTGAGACGTTATGTAAAGCACAATCATTTAGATGAAGGGATGATAACGAGA 87

SmTAL5_Smp_195090 AGAGATGAACTGAGACGTTATGTAAAACATAATCATTTAGATGAAGGGATGATAACGAGA 120

ShTAL5_v1_MS3_05957 AGAGATGAATTGAGACGTTATGTAAAGCACAATCATTTAGATGAAGGGATGATAACGAGA 120

ShTAL5_expression_vector AGAGATGAATTGAGACGTTATGTAAAGCACAATCATTTAGATGAAGGGATGATAACGAGA 120

********* ****************.** ******************************

ShTAL5_v3_MS3_00002091 TGGCAAGCATTATTTGATCCAACAAATACTGGTATTATAACATTTCAAAAATTTTGTGAT 147

SmTAL5_Smp_195090 TGGCAATCATTATTCGATCCAACAAATAGTGGTATTATAACATTTCAAAAATTTTGTGAT 180

ShTAL5_v1_MS3_05957 TGGCAAGCATTATTTGATCCAACAAATACTGGTATTATAACATTTCAAAAATTTTGTGAT 180

ShTAL5_expression_vector TGGCAAGCATTATTTGATCCAACAAATACTGGTATTATAACATTTCAAAAATTTTGTGAT 180

****** ******* ************* *******************************

ShTAL5_v3_MS3_00002091 GTCCTCGGTGTAAAACCGGAACAAGCACGTACTCTTCGGAAGAGTGTTGTCAATAACCGA 207

SmTAL5_Smp_195090 GTCCTCGGTGTCAAACCGGAACAAGCACGTACTCTTCGGAAGAGTGTTATCAATAACCGA 240

ShTAL5_v1_MS3_05957 GTCCTCGGTGTAAAACCGGAACAAGCACGTACTCTTCGGAAGAGTGTTGTCAATAACCGA 240

ShTAL5_expression_vector GTCCTCGGTGTAAAACCGGAACAAGCACGTACTCTTCGGAAGAGTGTTGTCAATAACCGA 240

***********.************************************.***********

ShTAL5_v3_MS3_00002091 CCACTGCCGAAAGATTTACAAATAATCTCCCAAAATATGTCACCAGAAGATCAATTCCAA 267

SmTAL5_Smp_195090 CCACTGCCGAAAGATTTACAAATAATCTCACAAAATATGTCATCAGAAGATCAATTCCAA 300

ShTAL5_v1_MS3_05957 CCACTGCCGAAAGATTTACAAATAATCTCCCAAAATATGTCACCAGAAGATCAATTCCAA 300

ShTAL5_expression_vector CCACTGCCGAAAGATTTACAAATAATCTCCCAAAATATGTCACCAGAAGATCAATTCCAA 300

*****************************.************ *****************

ShTAL5_v3_MS3_00002091 ATATTTGAATTTGTTCGATCACTGTTAGATAAAAATTTATCAGGTCAAGATATGACACAA 327

SmTAL5_Smp_195090 ATATTTGAATTTGTTCGATCATTGTTAGATAAAAATTTATCAGTTCAAGATATGACACAA 360

ShTAL5_v1_MS3_05957 ATATTTGAATTTGTTCGATCACTGTTAGATAAAAATTTATCAGGTCAAGATATGACACAA 360

ShTAL5_expression_vector ATATTTGAATTTGTTCGATCACTGTTAGATAAAAATTTATCAGGTCAAGATATGACACAA 360

********************* ********************* ****************

ShTAL5_v3_MS3_00002091 ATGATAAAACAATGGCTTGATAAGACATTCGACCCTTCATGGCATGTTGTTATAATCGAT 387

SmTAL5_Smp_195090 ATGATAAAACAATGGCTTGATAAGACATTTGACCCTTCATGGCATGTTGTTATAATCGAT 420

ShTAL5_v1_MS3_05957 ATGATAAAACAATGGCTTGATAAGACATTCGACCCTTCATGGCATGTTGTTATAATCGAT 420

ShTAL5_expression_vector ATGATAAAACAATGGCTTGATAAGACATTCGACCCTTCATGGCATGTTGTTATAATCGAT 420

***************************** ******************************

ShTAL5_v3_MS3_00002091 GGTTCATATTGGATATCGTATTCACATTTACCTGAACAATCTCTACAATTTCGATTGAAA 447

SmTAL5_Smp_195090 GGCTCATATTGGATATCATATTCACATTTACCTGAACAATCTTTACAATTTCGATTGAAA 480

ShTAL5_v1_MS3_05957 GGTTCATATTGGATATCGTATTCACATTTACCTGAACAATCTCTACAATTTCGATTGAAA 480

ShTAL5_expression_vector GGTTCATATTGGATATCGTATTCACATTTACCTGAACAATCTCTACAATTTCGATTGAAA 480

** **************.************************ *****************

ShTAL5_v3_MS3_00002091 GAAAAGTGTTATTTATTGTCTACGCAAAATACTTCGGATCCATTGGCCAGACACCATCTG 507

SmTAL5_Smp_195090 GAAAAGTGTTATTTAGTAT-GGCGCACAC-----CTA-AACATTGA-------------- 519

ShTAL5_v1_MS3_05957 GAAAAGTGTTATTTAGTTT-GGCGCACAC-----CTA-AATATTGA-------------- 519

ShTAL5_expression_vector GAAAAGTGTTATTTAGTTT-GGCGCACAC-----CTA-AATATTGA-------------- 519

*************** * * .****.*. * . :. ****.

ShTAL5_v3_MS3_00002091 CAACAACCTACTGTAAGAGAGAAGAAACTAGATCCCAGAGGAGGAAGAAATCAGGAAGAA 567

SmTAL5_Smp_195090 ------------------------------------------------------------ 519

ShTAL5_v1_MS3_05957 ------------------------------------------------------------ 519

ShTAL5_expression_vector ------------------------------------------------------------ 519

Multiple sequence alignment of ShTAL6 version 1 and ShTAL5 version 3 genome annotation cDNA sequences with SmTAL6, performed in CLUSTAL O (1.2.4). ShTAL5 V3.0 sequence truncated, showing nucleotides 1201–1697 only.

SmTAL6_Smp_072620 -------------------------------ATGTTTAAT----GTTTGTAATTTTTCCA 25

ShTAL5_v3_MS3_00002091 TTCGCCAAAGAGAATCATCTGGGTAATCGAATGGTTCAAAGATGGTTTGAGCTATTTTCG 1260

ShTAL6_v1_MS3_05176 ------------------------------ATGGTTCAAAGATGGTTTGAGCTATTTTCG 30

: *** **: *****:..*:*** *.

SmTAL6_Smp_072620 CTAATGTCATATGTACCAGTATCATTCTTCTCTTAATCCATACAAACCAT--ACTTTTAA 83

ShTAL5_v3_MS3_00002091 GAAGAGACAACAA-AC------CAAATTACACTTAATA---------------------- 1291

ShTAL6_v1_MS3_05176 GAAGAGACAACAA-AC------CAAATTACACTTAATAAGTTTTTAAGTGTTCTAGGTGT 83

:*.:*:**:.:. ** **:: *:*:******.

SmTAL6_Smp_072620 TATATATTTTTATAGTGAAATAATACGAAGAAATACAACCCAACAGAATGCAGCATTGTT 143

ShTAL5_v3_MS3_00002091 --------------ATGAAAAAATGCGAAGAAATACAATTCAACAACATTCAGCATTGTT 1337

ShTAL6_v1_MS3_05176 TGCAAAAGAGGAATATGAAAAAATGCGAAGAAATACAATTCAACAACATTCAGCATTGTT 143

.*****:***.************* *****..** **********

SmTAL6_Smp_072620 TAAACTTGGTTCAGATATTGAATACATTTCTGGTGATATGATGTTATCACAACAAATCAA 203

ShTAL5_v3_MS3_00002091 TAAACTTGGCTCAGACATTGAATACATTTCTGGCGATATGATGTTACCACAACAAATCAA 1397

ShTAL6_v1_MS3_05176 TAAACTTGGCTCAGACATTGAATACATTTCTGGCGATATGATGTTACCACAACAAATCAA 203

********* ***** ***************** ************ *************

SmTAL6_Smp_072620 TGTCACCAATGAGGCTAGAAAATTATATCAAGAATATAAATCTGAAAATAAGCTGTTGAT 263

ShTAL5_v3_MS3_00002091 TGTCAGCAATGAAGCCAGAAAATTATATCAAGAATATGAATGCGACAATAAAATATCGAT 1457

ShTAL6_v1_MS3_05176 TGTCAGCAATGAAGCCAGAAAATTATATCAAGAATATGAATGCGACAATAAAATATCGAT 263

***** ******.** *********************.*** **.*****..*.* ***

SmTAL6_Smp_072620 TGCTACAAAGCTTAAAGAATTTCTAGATAAAACATTTGGCAGAGCGTGGCATGTTACGGT 323

ShTAL5_v3_MS3_00002091 TGCTACAAAGCTTAAAGAATTTCTAGATAGAGCATTTGGTAGGTCATGGCATGTTACGGT 1517

ShTAL6_v1_MS3_05176 TGCTACAAAGCTTAAAGAATTTCTAGATAGAGCATTTGGTAGGTCATGGCATGTTACGGT 323

*****************************.*.******* **. *.**************

SmTAL6_Smp_072620 GGTGGATGGTTCGTTTGCCAGTGCACATACAGAAGAAGTTAATACTTCATTCCATTTCAA 383

ShTAL5_v3_MS3_00002091 AGTGGATGGTTCCTTTGCCAGTTCATATACACAAGAAGTTAATACTTCATTTCACTTTAA 1577

ShTAL6_v1_MS3_05176 AGTGGATGGTTCCTTTGCCAGTTCATATACACAAGAAGTTAATACTTCATTTCACTTTAA 383

.*********** ********* ** ***** ******************* ** ** **

SmTAL6_Smp_072620 AATGAAAAATCTTTGTTTTATTATATGGAAAACACCAGATTGTAGAAATGAATAA----- 438

ShTAL5_v3_MS3_00002091 AATGAAAAATCTTTGTTATCTTATATGGAAAACACCAGAGTACATAGATGAATAATTGAT 1637

ShTAL6_v1_MS3_05176 AATGAAAAATCTTTGTTATCTTATATGGAAAACACCAGAGTACATAGATGAATAA----- 438

*****************:*.******************* *. * *.********

SmTAL6_Smp_072620 ------------------------------------------------------------ 438

ShTAL5_v3_MS3_00002091 AAAATGATTTCAGTAATAAATGTATTCAAGCAGTAAAAACGTGTTTTTAAATATTCAAAG 1697

ShTAL6_v1_MS3_05176 ------------------------------------------------------------ 438

**Fig A**. **Lifecycle transcription profiles of *S. haematobium* TAL proteins** (Data from Stroehlein *et al*. (1)).

**Fig B**. **Lifecycle transcription profiles of *S. mansoni* TAL proteins** (Data from meta-analysis by Lu *et al.* (2)).

**Gel electrophoresis of ShTAL proteins**

Recombinant TAL proteins of an appropriate molecular weight were expressed in an *E. coli* expression system.


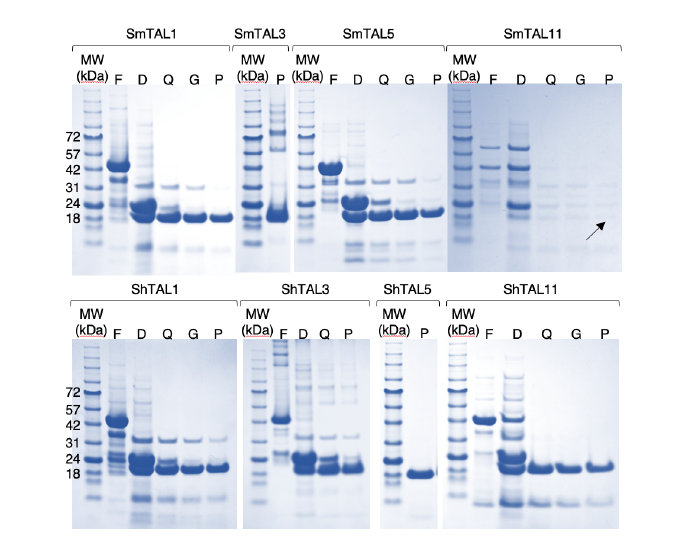


**Fig C.** **Gel electrophoresis of purified SmTAL and ShTAL proteins**. Coomassie blue-stained SDS-PAGE gel of recombinant *S. mansoni* and *S. haematobium* TAL1, TAL3, Tal5 and TAL11, including fusion protein (F); thrombin digest (D); and subsequent purification steps (Q: Q-sepharose bead purification; G: glutathione Sepharose bead purification; P: purified protein following aminobenzamidine agarose bead purification.

**Fig D. Pre- and post-treatment (Rx) seroprevalence of IgE responses to *S. haematobium* TAL proteins by age and village, as a proxy for force of transmission.** Error bars represent 95% confidence intervals around the seroprevalence. Children represent individuals less that 10 years old; ‘adults’ represent individuals aged 10 years and older, based on age of peak CAA across whole cohort. Number of individuals within each demographic group: Children (n = 70) and adults (n = 88) in moderate intensity village; Children (n = 32) and adults (n = 71) in high intensity villages.

**Table A. Recombinant antigen coating concentration**

| **Antigen** | **Stock concentration (mg/ml)** | **Coating dilution** |
| --- | --- | --- |
| SmTAL1 | 0.87 | 1:100 |
| SmTAL3 | 0.70 | 1:100 |
| SmTAL5 | 0.66 | 1:100 |
| SmTAL11 | 0.21 | 1:40 |
| ShTAL1 | 0.42 | 1:80 |
| ShTAL3 | 0.48 | 1:80 |
| ShTAL5 | 0.36 | 1:100 |
| ShTAL11 | 0.57 | 1:100 |

**Table B**. **Percent identity matrix between ShTAL1, 3, 5 and 11 and SmTAL1, 3, 5 and 11.** Alignment performed in CLUSTAL O (1.2.4)

|  | ShTAL1 | ShTAL3 | ShTAL5 | ShTAL11 |
| --- | --- | --- | --- | --- |
| ShTAL1 | 100.00 | 32.43 | 33.72 | 33.52 |
| ShTAL3 | 32.43 | 100.00 | 45.35 | 43.18 |
| ShTAL5 | 33.72 | 45.35 | 100.00 | 46.51 |
| ShTAL11 | 33.52 | 43.18 | 46.51 | 100.00 |
|  | SmTAL1 | SmTAL3 | SmTAL5 | SmTAL11 |
| SmTAL1 | 100.00 | 29.31 | 30.59 | 26.44 |
| SmTAL3 | 29.31 | 100.00 | 44.77 | 40.56 |
| SmTAL5 | 30.59 | 44.77 | 100.00 | 44.19 |
| SmTAL11 | 26.44 | 40.56 | 44.19 | 100.00 |

| **Table C. Association between pre-treatment ShTAL-specific IgE responses (seropositivity) and baseline CAA, as a measure of worm burden**. Results for the reduced linear regression model, adjusted for age and village are displayed, for total cohort (n = 261). | | | | | | | | | | | |
| --- | --- | --- | --- | --- | --- | --- | --- | --- | --- | --- | --- |
|  | **ShTAL1** | | | **ShTAL3** | | **ShTAL5** | | | **ShTAL11** | | |
|  | **GM ratio (95%CI)** | **P-value** | **GM ratio (95%CI)** | | **P-value** | **GM ratio (95%CI)** | **P-value** | **GM ratio (95%CI)** | | **P-value** |  |
| Age (years) ^†^ |  |  |  | |  |  |  |  | |  |  |
| 7–9 | 11.48 (3.91–33.74) | *** | 12.56 (4.31–36.62) | | *** | 11.4 (4.09–34.80) | *** | 11.95 (4.11–34.71) | | *** |  |
| 10–14 | 33.83 (10.16–112.7) | *** | 36.46 (10.93–121.6) | | *** | 33.75 (10.08–112.9) | *** | 33.12 (9.97–110.0) | | *** |  |
| 15 – 24 | 25.99 (7.20–93.88) | *** | 29.05 (7.99–105.67) | | *** | 26.01 (7.18– 94.15) | *** | 26.30 (7.31–94.56) | | *** |  |
| 25 – 29 | 16.34 (5.38–49.60) | *** | 18.65 (6.19–56.23) | | *** | 16.98 (5.65–51.06) | *** | 15.93 (5.30–47.84) | | *** |  |
| 30+ | 10.18 (2.81–36.89) | *** | 11.34 (3.13–41.09) | | *** | 10.38 (2.87–37.58) | *** | 10.50 (2.92–37.75) | | *** |  |
| Village^‡^ | 175.0 (44.1–693.9) | *** | 225.01 (55.25–916.3) | | *** | 180.1 (45.49–712.9) | *** | 166.3 (42.19–655.3) | | *** |  |
| TALx-IgE^§^ | 1.19 (0.66–2.14) | ns | 0.67 (0.35–1.30) | | ns | 1.10 (0.57–2.13) | ns | 2.14 (0.70–6.56) | | ns |  |
| Interaction terms |  |  |  | |  |  |  |  | |  |  |
| 7–9 * village | 0.066 (0.010–0.45) | ** | 0.061 (0.009–0.41) | | ** | 0.061 (0.009–0.42) | ** | 0.055 (0.008–0.38) | | ** |  |
| 10–14 * village | 0.030 (0.004–0.21) | *** | 0.028 (0.004–0.19) | | *** | 0.030 (0.004–0.21) | *** | 0.030 (0.004–0.21) | | *** |  |
| 15 – 24 * village | 0.015 (0.002–0.11) | *** | 0.013 (0.002–0.09) | | *** | 0.014 (0.002–0.10) | *** | 0.015 (0.002–0.11) | | *** |  |
| 25 – 29 * village | 0.008 (0.001–0.05) | *** | 0.007 (0.001–0.04) | | *** | 0.008 (0.001–0.05) | *** | 0.009 (0.001–0.06) | | *** |  |
| 30+ * village | 0.008 (0.001–0.07) | *** | 0.007 (0.001–0.06) | | *** | 0.008 (0.001–0.08) | *** | 0.007 (0.001–0.07) | | *** |  |
| ***p<0.001, **p<0.01, *p<0.05, ^.^ p<0.1, ns non-significant; ^†^ values are compared to 5- to 6-year-old age group; ^‡^ values are for high intensity villages compared to moderate infection intensity village; ^§^ ﻿where TALx represents the indicated combination of TAL1, TAL3, TAL5 and TAL11 responses. | | | | | | | | | | | |

| **Table D. Association between pre-treatment ShTAL-specific IgE responses (seropositivity) and baseline CAA, as a measure of worm burden**. Results for the reduced linear regression model, adjusted for age, sex, village and ShTAL-specific IgG_4_ seropositivity are displayed, for total cohort (n = 261). | | | | | | | | |
| --- | --- | --- | --- | --- | --- | --- | --- | --- |
|  | **ShTAL1** | | **ShTAL1, 3** | | **ShTAL1, 3, 5** | | **ShTAL1, 3, 5, 11** | |
|  | **GM ratio (95%CI)** | **P-value** | **GM ratio (95%CI)** | **P-value** | **GM ratio (95%CI)** | **P-value** | **GM ratio (95%CI)** | **P-value** |
| Age (years) ^†^ |  |  |  |  |  |  |  |  |
| 7–9 | 4.70 (1.86–11.89) | ** | 4.65 (1.87–11.59) | ** | 4.64 (1.88–11.48) | *** | 4.09 (1.65–10.13) | ** |
| 10–14 | 8.84 (3.29–23.73) | *** | 8.86 (3.38–23.26) | *** | 9.65 (3.69–25.21) | *** | 8.54 (3.27–22.28) | *** |
| 15 – 24 | 4.97 (1.82–13.56) | ** | 4.63 (1.71–12.48) | ** | 5.33 (1.97–14.41) | ** | 4.24 (1.53–11.74) | ** |
| 25 – 29 | 3.23 (1.27–8.17) | * | 2.68 (1.07–6.73) | * | 3.00 (1.20–7.52) | * | 2.86 (1.15–7.12) | ** |
| 30+ | 2.02 (0.67–6.12) | ns | 1.90 (0.64–5.67) | ns | 2.54 (0.84–7.72) | . | 2.40 (0.80–7.22) | ns |
| Sex | - | ns | - | ns | - | ns | - | ns |
| Village^‡^ | 5.03 (2.66–9.50) | *** | 4.09 (2.09–8.00) | *** | 3.34 (1.69–6.59) | *** | 2.86 (1.45–5.64) | ** |
| TALx-IgE^§^ | 1.06 (0.57–1.96) | ns | 0.59 (0.28–1.25) | ns | 0.54 (0.22–1.28) | ns | 1.02 (0.28–3.73) | ns |
| TAL1-IgG_4_ | 2.76 (1.37–5.62) | ** | - | ns | - | ns | - | ns |
| TAL3-IgG_4_ |  |  | 4.49 (2.22–9.08) | *** | 2.87 (1.29–6.45) | * | - | ns |
| TAL5-IgG_4_ |  |  |  |  | 3.03 (1.19–7.71) | * | 2.98 (1.23–7.24) | ***** |
| TAL11-IgG_4_ |  |  |  |  |  |  | 2.97 (1.43–6.17) | ****** |
| ***p<0.001, **p<0.01, *p<0.05, ^.^ p<0.1, ns non-significant; ^†^ values are compared to 5- to 6-year-old age group; ^‡^ values are for high intensity villages compared to moderate infection intensity village; ^§^ ﻿where TALx represents the indicated combination of TAL1, TAL3, TAL5 and TAL11 responses. | | | | | | | | |

**References**

1. Stroehlein AJ, Korhonen PK, Lee VV, Ralph SA, Mentink-Kane M, You H, et al. Chromosome-level genome of Schistosoma haematobium underpins genome-wide explorations of molecular variation. PLoS Pathog. 2022;18(2):e1010288.
2. Lu Z, Sessler F, Holroyd N, Hahnel S, Quack T, Berriman M, et al. A gene expression atlas of adult Schistosoma mansoni and their gonads. Sci Data [Internet]. 2017;4(1):170118. Available from: https://doi.org/10.1038/sdata.2017.118.
